# How to Mitigate the Hard Problem by Adopting the Dual Theory of Phenomenal Consciousness

**DOI:** 10.3389/fpsyg.2019.02837

**Published:** 2019-12-17

**Authors:** Michal Polák, Tomáš Marvan

**Affiliations:** ^1^Department of Philosophy, Faculty of Philosophy and Arts, University of West Bohemia, Pilsen, Czechia; ^2^Department of Analytic Philosophy, The Czech Academy of Sciences, Institute of Philosophy, Prague, Czechia

**Keywords:** hard problem, consciousness, phenomenality, phenomenal consciousness, unconscious phenomenality

## Abstract

In this paper, we propose the following hypothesis: the hard problem of consciousness is in part an artifact of what we call the unitary approach to phenomenal consciousness. The defining mark of the unitary approach is that it views consciousness and phenomenality as inseparable. Giving up this conceptual commitment redefines, in a productive way, the explanatory tasks of the theory of consciousness. Adopting a non-unitary conception of experience does not make the hard problem of consciousness go away completely but it shifts the locus of where the explanation of experience gets difficult, and cuts down the mystery of consciousness to size. Other advantages of the non-unitary account of consciousness are sketched as well.

## Introduction

The hard problem of consciousness^1^ arises due to the unavailability of convincing answers to the question as to how material information-processing events in the brain give rise to conscious phenomenal experience (Chalmers, 1996, 1998). Extensive literature now exists detailing the various proposed ways of dealing with the hard problem (see, e.g., the essays collected in Shear, 1999, and Chalmers’ responses in this volume, or the systematic survey in Block, 2003). The solutions offered range from saying that there is no hard problem (Dennett, 2018) or that its existence is a persistent illusion that needs to be explained (see the articles collected in Frankish, 2017), to claiming that the problem does exist and is completely intractable (McGinn, 1989). A less radical strategy is to accept the challenge posed by the hard problem but then show that once one makes certain theoretical move(s), the hard problem stops giving the impression of being intractable. We might call the family of such explanatory proposals “mitigating strategies.” Robinson (1996), to give one example, promotes a version of mitigating strategy. He draws attention to the fact that our current conceptual framework for theorizing about consciousness is based on structural and relational explanations. Qualitative properties, however, are standardly supposed to be non-structural and non-relational properties. Thus, he concludes, we can never get non-structural and non-relational explanation of the hard problem within structural and relational conceptual framework. The hard problem might be more tractable in a different explanatory framework.

Chalmers (2018) contains the most recent discussion of a different mitigating strategy: the acceptance of unconscious sensory qualities. According to Chalmers, such a move, due primarily to Rosenthal (2005c), is

perhaps most promising for deflating the explanatory gap tied to qualities such as redness: if these qualities […] can occur unconsciously, they pose less of a gap. As before, however, the core of the hard problem is posed not by the qualities themselves but by our *experience* of these qualities: roughly, the distinctive phenomenal way in which we represent the qualities or are conscious of them. (Chalmers, 2018, p. 30).

We believe that this mitigating strategy could prove to be more promising than Chalmers is ready to admit. It all depends on how we understand the idea of unconscious sensory qualities.

Before we proceed toward expounding our mitigating strategy, a couple of words should be said about the hard problem and about how we propose to approach it. The general consensus is that the hard problem is about phenomenal aspects of conscious experience. The worry is that these will remain beyond the reach of empirical science. What constantly fuels this worry, we believe, is taking the conscious subjective phenomenal experience to be something monolithic. The peculiar nature of subjective qualities and their being conscious comes as a package and it is difficult to conceive how science might begin explaining it. One way of proceeding is to decide that only a completely revolutionary twist within science (e.g., the discovery of completely novel natural laws explaining the emergence of conscious experience) will be of any real use here. We do not wish to exclude such a possibility out of hand. But pending a clear idea of how this is to be done – and nobody today has such an idea – the most reasonable alternative seems to be taking the piecemeal approach. The signature theoretical maneuver of this approach is *decomposition*. The conscious subjective experience is being felt as something unitary, we grant that. But that does not mean that if we look behind the subjective level and try to explain how such unitary experience arises, the explanation itself has to have unitary form. What if we start by peeling off an important aspect of the hard problem and relegate it to the category of the more tractable problems of consciousness, amenable to the standard methods of science? And what if, not stopping there, we could peel off, one after another, the remaining aspects of experience? By decomposing the processes forming conscious phenomenal experience, we allow for the piecemeal, gradual explanation of its constitution. Perhaps by doing this systematically, one day we will conclude that we are finally able to explain a sufficient number of such aspects – and the hard problem will be gone for good.

Our attempt is of this sort. We are aiming to decompose the concept of conscious phenomenal quality in its more fundamental parts, hoping that the result is a step toward mitigation of the hard problem. These more fundamental parts, we propose, are phenomenal quality on the one hand and the process of making this quality conscious, on the other. Based on this decomposition, further ones can be launched in the future.

## The Default Assumption of Contemporary Consciousness Studies

Chalmers seems to accept the possibility of unconscious sensory qualities but does not think that the hard problem could be mitigated by such an admission. Clearly, as he conceives them, unconscious sensory qualities are completely devoid of phenomenal aspects: their phenomenality is restricted to the conscious level.^2^ The view that phenomenality is conscious by definition might be called the default assumption of contemporary consciousness studies. We believe that this assumption blocks progress in thinking about the hard problem and needs to be revisited.

The default assumption that consciousness and phenomenality are inseparable is a cornerstone of what might be called the unitary view of phenomenal consciousness. It is unitary because consciousness and phenomenality are united in one experience and it is not possible to separate them. An alternative conception, the *dual* view of phenomenal consciousness, puts forward two independent claims: (1) phenomenality and consciousness are not inextricable, (2) non-conscious phenomenality is possible. In this view, the experiential “what-it’s-like-ness” of a conscious mental state is a result of the interaction between the phenomenal character of a mental state and the process of making this character conscious. From the neuroscientific point of view, the dual models allow to disentangle the neural processes in which the phenomenal quality is formed from the processes that make this quality conscious. The dual hypothesis opens up a plausible possibility that conscious and non-conscious mental states are episodes of the same kind, while acknowledging their undeniable differences. The sameness consists in that both non-conscious and conscious states can share their phenomenal character. The difference resides exclusively in their being consciously apprehended or not.

There is a host of compelling yet independent arguments for the dual approach. In Marvan and Polák (2017), we classified them into four categories: conceptual, methodological, neuropsychological, and neuroscientific. Let us summarize them very briefly without getting into fine details. The conceptual argument is inspired by the ideas of Rosenthal (1986, 2011). The argument is simple: conceptually, it makes good sense to postulate unconscious but qualitative mental properties. Rosenthal picks pain as an example. He points out that a person can be in a state of pain for a longer period of time without being aware of her pain for this whole period. It might be claimed that even in the periods in which she is distracted by other, more attention-consuming stimuli that disallow her to feel her pain consciously, she is still in the state of pain. All that the conceptual argument intends to prove is that it is not conceptually incoherent to speak about unconscious pains and other such unconscious qualitative states occurring within out-of-awareness periods. Although this argument cannot prove the correctness of the dual model separating phenomenality and consciousness, it paves the way for it.

The methodological argument for the dual view draws on the maxim that empirical research must treat consciousness of a mental state as an experimental variable. The presence or absence of consciousness should, then, be the sole distinguishing criterion of otherwise identical states. Applied to the case of phenomenal consciousness, this standard methodological desideratum demands that the scientists study pairs of phenomenal mental states of which one is and the other is not conscious, the presence/absence of consciousness being their sole distinguishing feature. The dual model satisfies this requirement, the unitary model does not. In the unitary model, conscious and unconscious mental states are different in more than one feature: conscious mental states have both phenomenality and consciousness, while unconscious mental states are missing both equally. For methodological reasons, then, the dual view is to be preferred to the unitary one.

The neuropsychological argument purports to show that we can use the assumption of unconscious phenomenal states to account for discriminatory and other behavioral abilities of neuropsychological subjects. Unilateral visual neglect, the inability to see objects in one half of the visual field, might serve as an illustration. In the most famous neglect example (Marshall and Halligan, 1988), a person cannot consciously discriminate between two depicted houses. The houses are identical except that one of them is on fire in that half of the visual field the person, due to neglect, cannot see. Although the person was constantly claiming that both houses look the same to her, she repeatedly said she would prefer to live in the house not consumed by the flames. The absence of conscious phenomenal information in one half of the visual field therefore does not preclude the brain to unconsciously process the stimuli present in this part of the field. The flames were somehow registered, though not consciously, and were driving the rational response of the patient. The dual view accounts for this situation in the following way: a full-blown phenomenal picture of the house was formed, was driving the behavioral reaction, but did not reach consciousness.^3^

The fourth type of argument for the dual view is that the view is perfectly compatible with some promising recent neuroscientific theories of consciousness. These theories permit clean separation of the brain mechanisms for the creation of phenomenal content from the mechanism that pushes this content into consciousness. Let us take Lamme’s (2006, 2015) theory as an illustration. The theory revolves around the notion of local recurrent neural activity within the cortex and decomposes the formation of conscious visual content into two phases. The first one is called fast feedforward sweep. It is a gradual activation of different parts of the visual system in the brain. The dual view interprets this process as the formation of the unconscious but phenomenal mental state. A later process, that may or may not occur, is called recurrent activity. It is a neural feedback processing during which higher visual centers send the neural signal back to the lower ones. The time delay between the initiation of the first and the second process might be seen as corresponding to the difference between processing of the phenomenal character (feedforward sweep) and making and maintaining this phenomenal character conscious (recurrent processing). This is by no means the only empirical model of consciousness that could support the distinction between the creation of phenomenal content and its entry into consciousness. Other theories such as Global Neural Workspace theory (Dehaene, 2014), thalamocortical circuits (Llinás et al., 1998), or apical amplification within the pyramidal neurons in cortex (Phillips et al., 2018; Aru et al., 2019) may serve as full-fledged neurobiological alternatives. In all these theories, the phase of phenomenal content creation and the phase of this content becoming conscious are clearly distinguishable.

In spite of this, the dual theory has virtually no advocates. Cognate ideas can be found in Wilkes (1984, 234f.); Rosenthal (2005a,b); Platchias (2011, ch. 3); Young et al. (2014); and Keller (2016), but it is not clear that any of those authors would accept the dual theory as we introduced it in the previous paragraphs. For instance, to see whether Rosenthal supports the dual view or not, one first needs to determine how to interpret his idea of unconscious sensory qualities. And while a dual reading of Rosenthal could be constructed from some textual evidence (see Rosenthal, 2005b,c, pp. 23, 32, and 135), most authors, including Chalmers in the quoted passage, take his unconscious sensory qualities to have no phenomenal features. The only philosopher who seems firmly committed to the dual approach is Coleman (2019).

The very idea of non-conscious phenomenal qualities is met with fierce protests because most authors take the impossibility of a non-conscious phenomenal character to be an incontrovertible *conceptual truth* about consciousness. This resistance is surprising given that arguments for the unitary framework are feeble. Its defense boils down to a purely terminological decision to confine phenomenality to the conscious level. The widely held identification of consciousness with phenomenal character, and the ensuing denial of non-conscious phenomenality, is not a result of evidence-based argument. The stubborn intuition that only conscious mental states can possess phenomenal character is probably based on the fact that we come to know the phenomenal character of mental states from instances of conscious perception and other kinds of conscious mental states and processes. But that does not license the inference that phenomenal character could not exist without consciousness.

As the reader probably noticed, we are using the terminology in an unorthodox way, proposing a reconceptualization of the notion of the “phenomenal.” But is not “unconscious phenomenality” an oxymoron? We believe not. Our choice of words is meant to reflect our theoretical commitments. The aim of this terminological choice is to stress that in their qualitative aspect, conscious and unconscious sensory features of a mental state can be identical. If we say, as is common, that unconscious mental states have sensory character but only the conscious mental states have phenomenal character, then that suggests that the latter differs from the former not just in being conscious for the subject but also in other, yet to be specified aspects. We think that the sensory aspects can be fully established in all their qualitative, i.e., phenomenal aspects already at the unconscious level. What is wholly located at the conscious level is only the mental state’s *what-it’s-like-ness*; our intuition is that this term cannot be stretched to the unconscious level. To be in a completely phenomenally unconscious state does not feel like anything.

This is not the place to defend the idea of non-conscious phenomenal qualities in depth, though. Our aim in this short contribution is only to indicate how the dual approach can be used to mitigate the hardness of the hard problem of consciousness. Chalmers (2018, p. 30) believes that placing the sensory qualities into the non-conscious part of the mind will not help to diminish the hard problem, because it is the *conscious experience* of these qualities that creates the gap between brain states and phenomenal states. But notice that this way of looking at things subscribes to the unitary framework. Chalmers’ refusal to accept that unconscious sensory qualities might help with the hard problem rests on the assumption that by switching into the conscious mode, mental states acquire a new, phenomenal form that was not there at the non-conscious level. In other words, Chalmers assumes that a substantial transformation takes place when sensory mental states become conscious. He is committed to hold that during the initial stages of perception the phenomenal character does not exist yet. There are only the non-phenomenal brain processes standing for features like shape, position in space, hue, motion, etc. (in the case of visual perception). As perception unfolds, phenomenality appears at the scene out of the blue, at the very same moment when consciousness arises. The same brain process creates both phenomenality and consciousness. As a consequence, experience might look like unfathomable mystery.

To better understand the difference between the standard, unitary framework for phenomenal consciousness and the dual proposal, it might be helpful to consider a view that lies somewhere in between them. In *Conscious Brain*, Prinz (2012, esp. pp. 126–145) suggests to distinguish the neural mechanisms establishing the phenomenal character from the mechanisms securing its being conscious. Phenomenal character is constituted by the neural processes called vectorwaves while the neural processes responsible for bringing this character into consciousness are synchronous gamma oscillations. These two processes are clearly distinguishable at the neural level, but, according to Prinz, they cannot appear separately. For a state to have its phenomenal character, both neural processes have to be activated and present at the same time. It follows that for phenomenal character to be present, consciousness has to be present as well. Having phenomenal character thus, on this theory, amounts to having *conscious* phenomenal character.

A consequence of this theory is that there is no unconscious phenomenal character. This, however, does not mean that there cannot be unconscious perception, such as in the cases of subliminal perception or masked priming (Prinz, 2012, p. 144). But the difference between conscious and unconscious perceptual states is precisely that the first have phenomenal character whereas the latter do not; phenomenal character is the very thing that distinguishes conscious from unconscious perception. In the regard that there is no phenomenality without consciousness, Prinz is therefore unitarian. On the other hand, his emphasis on the difference between mechanisms ensuring phenomenal qualities of perceptual experience and between mechanisms of consciousness is something that he shares with full-blooded dual theory. Thus, one might call his account of phenomenal consciousness “hybrid” for it combines the principles of both unitary and dual notion of consciousness.

The dual conception, in its pure form, assumes that mental contents do not acquire new form upon entering consciousness.^4^ For this reason, it has a potential to surpass the unitary conception in dealing with the hard problem. If we give up the unitary conception of phenomenal consciousness, the hard problem is stripped of its consciousness aspect and then shifted to a lower level. The question is no longer: in which way are the *conscious* phenomenal states produced in the underlying physical substrate but rather how can this substrate give rise to unconscious phenomenality. There will remain no special mystery of the difference between *conscious* phenomenal states and the underlying physical processes. In the dual perspective, becoming conscious of a mental state does not add anything special to the already formed phenomenal character of the state; it just makes it available for the subject. If we allow for the possibility of non-conscious states having phenomenal character, consciousness itself might ultimately transpire to be identifiable with a relatively simple neural mechanism. In particular, becoming conscious of independently constituted phenomenal qualities might perhaps be provided by a relatively simple process of neural recurrence/feedback/reentry (see, e.g., Tononi and Edelman, 1998; Lamme, 2015), or passing pre-formatted contents to the working memory (Prinz, 2012), or entry into the global neuronal workspace (Dehaene, 2014). All these processes might be seen as candidate mechanisms for the uptake of the phenomenal contents into consciousness. This simplifies the way we look at consciousness as such: it becomes underpinned by a single, non-changing neural mechanism, defined regardless of whether it uptakes cognitive, emotional, volitional, or sensory contents.

This neural mechanism, whatever its neural implementation might be in detail, does not have any conscious features by itself, but only due to the interaction with unconscious phenomenal features. In other words, consciousness asymmetrically depends on phenomenal qualities for its very existence, whereas phenomenal qualities can exist independently of consciousness. A remote possibility then arises of the mechanism for making phenomenal qualities conscious being active without any phenomenal contents being available to it. But this hypothetical possibility is harmless. Such empty “consciousness” would in fact feel like nothing for the subject whose consciousness mechanisms are so activated. Nothing inconsistent thus follows from accepting this sort of asymmetric dependence of consciousness on phenomenal qualities.

Tackled from the perspective of the dual approach, the problem of phenomenal consciousness stops giving the impression of being hard in Chalmers’ famous sense. The “mystery” of experience will not quite disappear, though, even in the dual framework. The difficult question surrounding the non-unitary approach is not the question of consciousness *per se* but the question of how the phenomenal character of a mental state gets constituted. No traditional hard problem *of consciousness* remains to be solved; but it continues to be unclear how the physical substrate can harbor phenomenal qualities. Some of the severity of the hard problem thus continues to surround the attempts to elucidate the processes that constitute phenomenality.^5^

Still, we believe that this is progress. The dual theory divides the problem of consciousness into the problem of explaining how the phenomenal character of a mental state is formed, and the problem of consciousness as such. Equipped with this distinction, the scientists can narrow the focus of research either into the mechanisms producing phenomenal character or into the processes constituting consciousness proper. The task of explaining phenomenal consciousness stops appearing so intractable because we are no longer facing the undifferentiated conglomerate of both consciousness and phenomenality.

A following objection might be voiced: splitting the problem of explaining consciousness into the search for two very different sorts of mechanisms might be a plausible strategy, but a strategy that does not really help with the hard problem. After all, one might claim, the hard problem is concerned with phenomenality from the start. We would find this objection a bit unfair. If you divide the hard problem into two separate problems and then show how one of them could in principle be solved, it is not fair to say that no progress has been made. To put it in terms familiar for consciousness researchers, one could also say that the dual view allows one to split the usually undifferentiated subjective experience into two aspects, and then to relegate the aspect of consciousness as such (i.e., the neural mechanism for making phenomenal states conscious) to the “easy” problems of consciousness, using Chalmers’ terminology. The consciousness as such might then be called “awareness.” Awareness in this sense is simply the process, describable in neuroscientific terms, of making the sensory qualities conscious for the subject.^6^ We could then keep using the term “consciousness” for the subjectively felt unitary experience, while holding that in reality this seemingly unitary thing is the result of an interaction between the neural processes constituting the phenomenal contents and the neural processes constituting awareness. We dare to claim that this strategy brings explanatory gains, although it does not make all of the hard problem go away (because the remaining task to explain phenomenality in strictly materialist terms remains challenging). But that is all right; we promised mitigation, not elimination of the hard problem.

## Further Virtues of the Dual Interpretation

The advantages of the dual conception are not limited to the promise of partial deflation of the hard problem. Another strength of the non-unitary explanation is that it accounts for non-conscious perception better than the unitary one. On the unitary view, non-conscious perception is composed of sophisticated neural processing but this processing entirely lacks phenomenal aspects. It follows that when we perceive non-consciously, we perceive *in a wholly different manner* than when we perceive consciously. When perceiving consciously, we rely on the phenomenal information. We distinguish between different colors, for instance, by means of the phenomenal information we are aware of. But how would a unitarian explain non-conscious perception? She cannot appeal to phenomenality. The only way left for her is to postulate two different sets of perceptual mechanisms, one for conscious perception with phenomenality and one for non-conscious perception without phenomenality.

We find this double-lined explanation not parsimonious. This problem does not beset the dual explanation. According to it, much the same perceptual discriminations can be performed consciously and non-consciously due to the shared phenomenal character – and shared neural mechanisms – of both conscious and non-conscious mental states. *If* conscious and non-conscious perceptual discriminations would systematically differ, one could assume that there are two different sets of perceptual mechanisms at work in conscious and non-conscious perception. A potent reason for supposing the existence of non-conscious phenomenal qualities would thus disappear. However, we know of no empirical work in psychophysics proving that conscious and non-conscious perceptual discriminations systematically differ.^7^

A related drawback of the unitary explanation is that every type of phenomenal state needs its own dedicated neural mechanism that makes it conscious. This is implied by the fact that for a unitarist, it is not possible to extricate the phenomenality aspect from the consciousness aspect of a mental state. Phenomenality and consciousness are the very same thing. Thus a neural mechanism for conscious perception of blue color is going to be somewhat different from the mechanism for conscious perception of green color; and the mechanism for conscious perception of color is going to be *quite* different from the mechanism securing conscious perception of sounds, smells, or pains. As a result, there cannot be an overarching unitary neuroscientific explanation of what makes different types of phenomenal states conscious. In reality, though, mental states are all conscious in the same way, only their phenomenal character varies. The dual explanation of phenomenal consciousness respects this intuition. It explicitly claims that there is a single, shared mechanism of consciousness across the board, working in tandem with different particular mechanisms for particular phenomenal contents.

Finally, the dual theory allows one to see that some competing theories of consciousness are addressing different fundamental questions. For instance, while the representationalist theory of consciousness (Tye, 1995) aims to say something illuminative about how the qualitative character of a mental state is constituted (as suggested by Prinz, 2012, pp. 19–20), the Higher Order Thought theory (Rosenthal, 2005a) and the Global Neuronal Workspace theory of consciousness (Dehaene et al., 2011; Dehaene, 2014) are mostly preoccupied with explaining how a mental state becomes conscious. Realizing that this is the case helps to ease the air of competition between such theories; they are simply working on different aspects of a single explanatory project.

## Concluding Remarks

We attempted on a mitigation of the mystery of the hard problem in the style of the dual conception of phenomenal consciousness. By separating phenomenality from consciousness as such, the dual framework clarifies the items in the space of explanations within consciousness studies, and their relations. According to the pure dual theory, conscious and non-conscious mental states are episodes of the same fundamental kind because they can share their phenomenal character. What differentiates between those two types of states might be a relatively simple neural process, the general neural correlate of awareness as such. What remains to be solved is the “hard problem” of the phenomenal character. This problem is hard but, we believe, not unsolvable.^8^

This way of conceiving things enables to split the empirical research of consciousness into the inquiry into the mechanisms of phenomenal character on the one hand and into the inquiry into the mechanisms of consciousness on the other. At the same time, the non-unitary approaches to phenomenal consciousness enable a more elegant and parsimonious explanation of how perception operates at both unconscious and conscious level, whereas on the dominant, unitary interpretation, this creates a mystery of its own.

The non-unitary models share the fundamental assumption that we need to dissociate the mechanisms providing for consciousness from the mechanisms generating the phenomenal character. We believe that this idea is under-appreciated both by philosophers of consciousness and also by empirical scientists. It should be explored in more detail. Of course, at the end of the day it may turn out that the dual interpretation is wrong and that one cannot mitigate the hard problem in the way we outlined. But if that happens, we will all be wiser in knowing that the theory of consciousness must be unitary. The acceptance of the unitary interpretation will no longer be based merely on entrenched intuitions.

## Author Contributions

Both authors listed have made a substantial, direct, equal and intellectual contribution to the work, and approved it for publication. They also agree to be accountable for all aspects of the work in ensuring that questions related to the accuracy or integrity of any part of the work are appropriately investigated and resolved.

### Conflict of Interest

The authors declare that the research was conducted in the absence of any commercial or financial relationships that could be construed as a potential conflict of interest.

## Footnotes

^1^We shorten “the hard problem of consciousness” to “the hard problem.”

^2^We use the terms “phenomenality” and “phenomenal character” as synonyms.

^3^Further, we have tried to show that the normal, non-pathological perception such as subliminal perceptual priming or visual masking can also serve as example of the severability of phenomenality and consciousness. For the full story, see Marvan and Polák (2017).

^4^The pure dual conception assumes that conscious and non-conscious phenomenal character are exactly the same. What remains to be adjudicated in future work is whether the dual theory-style uptake of a phenomenal character into consciousness may change or modify it somehow. If this is possible, the unconscious and conscious phenomenal character would no longer be the same. We are open to this possibility but we believe that the present state of scientific evidence allows one to defend the stronger, pure form of the dual conception.

^5^We should perhaps make clear that we take the phenomenal states to be a subclass of physical/material states. See Polák and Marvan (2018) and Jylkkä and Railo (2019).

^6^Note that this would actually come close to Chalmers’s desideratum (Chalmers, 1998, p. 11) to reserve the term “awareness” for a “less loaded” functional notion. But he defines awareness as having to do with discriminability, reportability, and other functional operations, whereas we think of awareness as a neural mechanism independent of the subject’s capabilities.

^7^Smallman et al. (1996) demonstrated that some extremely fine perceptual details cannot be perceived consciously but can be represented unconsciously. And He and MacLeod (2001) detected after-effects of fine grained visual stimuli that cannot be consciously distinguished from a uniform field. A possible interpretation of such findings is that unconscious phenomenality may, at least in some cases, be *richer* than the conscious one. This, though, does not impugn our main point, viz. that there is unconscious phenomenality and that it drives non-conscious perception.

^8^Much depends on what we expect the adequate explanation of phenomenal character to deliver. If the expectation is that the theory delivers an explanation with a deep subjective “sense of understanding,” the “Aha, I see it now” feeling (Trout, 2007), we might never be satisfied (see Wright, 2007).
